# Why statistical inference from clinical trials is likely to generate false and irreproducible results

**DOI:** 10.1186/s12874-017-0399-0

**Published:** 2017-08-22

**Authors:** Leonid Hanin

**Affiliations:** 1 Department of Mathematics and Statistics, Idaho State University, 921 S. 8th Avenue, Stop 8085, Pocatello, ID 83209-8085 USA; 2 Department of Applied Mathematics, Institute of Mathematics and Mechanics, St. Petersburg Polytechnic University, Polytekhnicheskaya ul. 29, 195251 St. Petersburg, Russia

**Keywords:** Clinical trial, Heterogeneity, *p*-value, Permutation test, Random sample, Randomization, Reproducibility, Sample size, Significance, Stochastic independence

## Abstract

One area of biomedical research where the replication crisis is most visible and consequential is clinical trials. Why do outcomes of so many clinical trials contradict each other? Why is the effectiveness of many drugs and other medical interventions so low? Why have prescription medications become the third leading cause of death in the US and Europe after cardiovascular diseases and cancer? In answering these questions, the main culprits identified so far have been various biases and conflicts of interest in planning, execution and analysis of clinical trials as well as reporting their outcomes. In this work, we take an in-depth look at statistical methodology used in planning clinical trials and analyzing trial data. We argue that this methodology is based on various questionable and empirically untestable assumptions, dubious approximations and arbitrary thresholds, and that it is deficient in many other respects. The most objectionable among these assumptions is that of distributional homogeneity of subjects’ responses to medical interventions. We analyze this and other assumptions both theoretically and through clinical examples. Our main conclusion is that even a totally unbiased, perfectly randomized, reliably blinded, and faithfully executed clinical trial may still generate false and irreproducible results. We also formulate a few recommendations for the improvement of the design and statistical methodology of clinical trials informed by our analysis.

## Background

Over the past several decades, biomedical sciences have made remarkable progress in understanding molecular and genomic mechanisms of life and disease. However, there remains an enormous gap between our understanding of life’s molecular machinery and our ability to explain behavior of living organisms as a whole and their responses to various interventions. In the medical arena, clinical trials aim to fill this gap using empirical means. One of the more dramatic manifestations of this gap is unexpected catastrophic events in early clinical trials. For example, a phase I trial of drug BIA 10-2474 aimed at treating anxiety, motor disorders and chronic pain that was conducted in 2015 in Rennes, France had unexpectedly led to the death of one volunteer and irreversible brain damage in five others [1]. In a phase I trial conducted in 2006 in London, a monoclonal antibody TGN1412 intended for treating autoimmune diseases and leukemia had caused multiple organ failure in six healthy volunteers [2]. As yet another example, fialuridine, an antiviral agent tested in 1993 by NIH for treatment of hepatitis B in a phase II clinical trial, had resulted in the death of five human volunteers due to severe hepatic toxicity and lactic acidosis [3]. Remarkably, the dose given to volunteers in the London trial was 500 times smaller than the one found to be safe in animals [4].

Insufficient knowledge of biological mechanisms and complex interactions associated with the action of drugs and other medical interventions creates a considerable uncertainty in predicting and interpreting trial outcomes. In particular, we are still unable to predict whether a tested intervention will work for an individual patient or a category of patients and what side effects it will produce. This causes the samples of trial participants to be heterogeneous in numerous unpredictable ways. Further, ethical considerations lead to recruitment of subjects that are generally younger, healthier and less medicated than the targeted population, which causes the sample of trial participants be not representative of the population. These uncertainties, combined with various biases and conflicts of interest involved in funding, planning, analyzing and reporting clinical trials, is a major reason behind surprisingly low effectiveness of many drugs and high incidence of severe side effects. As reported in [5], ten top-selling drugs on the American market fail to improve the conditions in 75% to 96% of patients who take them. Thus, lingering question remain as to whether even some highly prescribed drugs actually work. More ominously, prescription medicines have become the third leading cause of death in the US and Europe after cardiovascular diseases and cancer ([6], p. 1). Doubts about utility of clinical trials are not limited to the medical community; they are also circulating in the media [7].

On the academic front, the validity and reproducibility of biomedical research including clinical trials has been a matter of grave concern for more than two decades [8, 9]. As one striking example, a meta-analysis study [10] found that, out of 26 most highly cited reports of controlled randomized clinical trials that appeared in top medical journals, claimed positive effects of medical interventions and were later retested on larger groups of patients, 9 studies (35%) were either refuted or their claims of effect were found to be greatly exaggerated.

What are the root causes of falsity and irreproducibility of biomedical research findings? In an article titled “Why Most Published Research Findings are False” [11], John Ioannidis sought to explain this phenomenon. He assumed the following model of scientific discovery: several groups of investigators independently study a number of research questions by performing statistical analysis of empirical data. Each research finding has a prior probability to be true; however, this probability is modulated by random effects of statistical analysis with certain rates of false positive and false negative discoveries. A key model parameter is “bias,” defined as the probability to report as true a finding that was found by a research team to be false. The model leads to a Bayes-type formula for the probability of false discovery as a function of model parameters. Sample computations based on this formula have led the author of [11] to the conclusion encapsulated in the title of his paper.

An in-depth look at various instances of deliberate bias and institutional corruption in clinical trials conducted or sponsored by industry was taken by Peter Gøtsche in his remarkable book [6]. It documents numerous cases of withholding and falsifying data, selective reporting, suppressing information on adverse side effects, post hoc changes of endpoints, manipulation of patient inclusion criteria and study duration to achieve more favorable outcomes, intentional handicapping of comparators as well as an assortment of other unethical profit-driven activities. This has led to numerous major public health calamities. As one example, concealment and fabrication of trial data on cardiovascular side effects of COX-2 inhibitor Vioxx (rofecoxib), manufactured by Merck and marketed primarily as a NSAID painkiller, has caused about 120,000 deaths worldwide from 1999 to 2004 ([6], p. 161). Based on his extensive study, Gøtsche concluded that in the hands of Big Pharma clinical trials have become nothing more than marketing tools in disguise.

While the above-mentioned explanations are valid and instructive, they ignore perhaps the most critical component of any research study – the scientific methodology adopted by the research team. In this article, we focus exclusively on statistical methodology used for planning and analyzing phase III clinical trials. We show that statistical inference from even a totally unbiased, properly randomized, reliably blinded, perfectly executed and faithfully reported controlled clinical trial is still likely to generate false knowledge and irreproducible results. A number of specific problems associated with the design of clinical trials, statistical inference from trial data and assessment of trial quality were discussed in [12].

As a reminder and point of reference, we review very briefly the methodology of clinical trials, see e.g. [13]. Phase III clinical trials are conducted to make large-scale empirical comparisons of medical interventions (one of them normally being a placebo or standard treatment). Two arms of a trial are compared by computing the difference between the average values of a certain measure of effect over all subjects in the respective arm of the trial. A typical measure of effect for an individual subject is either the indicator of occurrence of certain event (response to treatment, cure, death, etc.) or the value of some observable clinical variable (e.g. disease-free survival time, systolic or diastolic blood pressure, count of some type of blood cells, concentration of certain biomarker, and so on). The normalized difference between the above averages usually serves as a test statistic employed for both trial planning and analysis of trial data.

Trials are designed in such a way that the null hypothesis of the treatments’ equal performance can be rejected with sufficiently low probability (called significance level) if they in fact perform equally and with high enough probability (called statistical power) if the alternative hypothesis that one treatment performs sufficiently better than the other is true. Normally, precautions against various biases are taken. In particular, patients are randomly assigned to treatments and, when feasible, patients and investigators are blinded to this assignment. After the trial’s completion, statistical analysis of its results is conducted to determine the actual significance level and power, estimate parameters of interest and compute confidence intervals, assess incidence and severity of side effects, etc.

Inherent in clinical trials are four principal sources of variation: (1) sampling of trial participants from a relevant population; (2) intra-subject (individual) variation in the effects of compared interventions; (3) between-subject variation in these effects; and (4) random assignment of trial participants to treatments. Only patients who meet the study entry criteria and give informed consent are enrolled in clinical trials. This and many other reasons cause sampling of subjects from the population to be non-random. This cannot be mitigated by even the best statistical analysis of trial data and thus is not a subject of this study. However, non-random sampling represents a major obstruction to extrapolation of trial findings to the population and their reproducibility. Variation types 2, 3 and 4 will be addressed in subsequent sections.

Our critique of statistical methods used in clinical trials proceeds along two dimensions. First, we identify several basic assumptions and principles underlying statistical methodology and argue that in the case of clinical trials their validity is uncertain, questionable or very likely false. Second, we address a few important technical aspects of statistical analysis commonly employed in clinical trials and conclude that they too are likely contributors to the generation of false and irreproducible results. Specifically, we start with the randomness vs determinism dilemma vis-à-vis individual response variables. Next, we discuss the averaging principle as a preference rule for selection of medical interventions. Then we take a close look at the fundamental assumptions of independence and homogeneity; we do so first by means of an example (also discussed in [14]) and then approach this topic from a more formal standpoint of statistical analysis. Further, we discuss individual case studies as an alternative to clinical trials. In the section Statistical Analysis of Clinical Trials as a Ritual, we review several statistical concepts and tools commonly employed in the analysis of trial data and argue that ignoring underlying assumptions, uncritical use of various approximations and arbitrary thresholds, and disregarding randomization may lead to false results. Finally, we summarize our findings, frame them in historical and philosophical perspectives, and formulate our conclusions and specific recommendations.

The presence of serious deficiencies in statistical methodology utilized in clinical trials does not mean that clinical trials should be abandoned. When carefully planned and properly conducted, they can produce a wealth of empirical knowledge about the disease and patients’ responses to the compared treatments. This may prove especially valuable when the disease and/or the effects or side effects of the tested interventions are very heterogeneous. However, for trials to be effective and inference from their results valid, statistical methodology should be considerably tightened, fortified with rigorous mathematical and computational sensitivity analyses, and combined with biomedical knowledge of the disease as well as biological and/or pharmacological action of the compared treatments.

## Individual response to treatment: The determinism vs chance dilemma

Nowadays, statistical analysis has become a mandatory component of biomedical research. This compliance pressure has caused biomedical scientists to adopt, mostly unwittingly, the assumption that every health-related event occurring in a given subject depends in essential ways on chance and that every measurable quantity is a random variable. That this assumption is not merely philosophical is clear: detection of the occurrence of a non-random event over the duration of a study is a matter of single observation while a random event occurs with certain probability whose estimation requires a large number of observations. It is an empirical fact that responses of different subjects to the same treatment display a wide variation. However, how strong is scientific evidence for the involvement of chance in *individual* responses?

The deterministic side of the dilemma enjoys a strong backing from basic science. The effects of drugs and other medical interventions typically manifest through the action of various biochemical systems that constitute the molecular basis of life. What we know about their functioning suggests that they essentially act as deterministic machines governed by differential equations of biochemical kinetics. If the initial concentrations of all molecular species, kinetic constants, and various external and internal conditions are known, then the future states of a biochemical system can be predicted with great accuracy. Such a predictable operation within a wide range of internal and external conditions is the reason why the genetic apparatus of a cell, which is nothing more than an extremely complex, self-regulating biochemical system, displays such a great fidelity in preservation and replication of the genome as well as in transcription, translation, and adaptive regulation of gene expression. Additional features of biochemical systems, such as activation thresholds and the presence of inhibitors and feedback loops, ensure stable execution of biological functions even in randomly changing environments, which contributes to physiologic homeostasis.

The deterministic behavior of biochemical systems is a collective result of a very large number of random microevents governed by stochastic laws of quantum mechanics. Therefore, before embarking on statistical analysis of individual response data one has to envision a mechanism by which stochasticity re-emerges, in the particular setting at hand, at the level of whole-body clinical effects. Or is this merely an illusion that masks unknown mechanisms of deterministic causation?

The hypothesis of deterministic response can sometimes be tested empirically. For example, if repeated occurrences of an acute illness in a given subject are cured by the same dose of a drug then the patient’s response to the drug is likely to be deterministic. Likewise, our casual experience with various drugs and medical procedures suggests that many of them have a stable, well-defined patient-specific effect in terms of both magnitude and timing. Why couldn’t this, then, be the case for an experimental drug tested in a clinical trial?

On the other hand, individual effects of certain treatments are undoubtedly stochastic. A classic example is exposure to radiation that can cause observable effects through cell killing, mutagenesis and carcinogenesis. Here, lethal damage to a cell or a harmful mutation can result from a random amount of energy deposited by a single particle of ionizing radiation, or one of its secondary particles, if the particle track happens to pass close enough to the cell’s DNA.

The choice between deterministic and stochastic approaches to mathematical or statistical modeling of individual effects of treatment should be deliberate and follow the preponderance of biomedical evidence regarding the nature of the disease and treatment. If such evidence is inconclusive then both approaches can be pursued competitively and the results compared.

## The methodology of clinical trials: Does averaging work?

The core methodological idea in clinical trials is the comparison of averages. The power of averaging lies in the combination of (1) essential cancellation of random individual variation and (2) retention of systematic clinical effects assumed to result from medical interventions. This idea, however, hinges on a hidden assumption of homogeneity, i.e. that the magnitude of the mean individual responses to the assigned treatment is about the same for most patients. The reality of most clinical trials, however, is very different. Typically, a sizeable fraction of subjects enrolled in a trial does not respond to the assigned treatment while responses of other subjects display a large variation in the magnitude and timing of the effect. Additionally, a wide variety of side effects ranging from minor and transient to permanent and life-threatening are observed.

The seemingly appealing idea that the best intervention is the one that works best on the average may be true in the case of homogeneous responses. However, as a general comparison principle, it represents a fundamental fallacy. What may appear best on the average may not be the best intervention even for a single patient in the population of interest. As a simple schematic example, suppose there are three competing drugs, A, B and C, compared on a population of patients. Let the efficacy of drug A be 2 units on a certain scale on one half of the population and 0 on the other half, and let drug B have efficacy of 2 on the latter half of the population and 0 on the former. Suppose drug C has efficacy of 1.1 across the board. Then drug C is superior to A and B on the average but for each particular patient it is almost twice less effective than the better of the drugs A or B!

To compare treatments based on their average responses, trialists have to minimize heterogeneity of the anticipated arm-specific individual responses at the planning stage of the trial. To this end, they are advised to use all available prior biomedical information about the targeted disease and mechanisms of drug action and adopt strict study entrance criteria.

## The heterogeneity curse: An example

To see how unreasonable is our reliance on the homogeneity of responses to a given treatment in clinical trials, consider a hypothetical randomized and appropriately blinded clinical trial that compares survival or metastasis-free survival of stage I-III breast cancer patients under two treatment plans involving surgery and various combinations and regimens of adjuvant cytotoxic chemotherapy, external beam radiation and hormonal therapy. What are the leading factors that determine individual survival outcomes? The single most important among them is the presence or absence of subclinical metastases at the time of surgery. Such metastases may be in three distinct states: (1) solitary cancer cells that were released into the bloodstream during surgery or were already present at the time of surgery as circulating tumor cells or quiescent cancer cells lodged at various secondary sites; (2) dormant or slowly growing avascular micrometastases; and (3) aggressively growing vascular secondary tumors that at the time of diagnosis have not yet reached detectable size. If a patient was metastasis-free at surgery then, barring primary tumor recurrence, she will be cured. If only state 1 and 2 metastases were present immediately after surgery then the outcome depends critically on how long the state of metastatic dormancy will be maintained. (For an extensive discussion of the significance of metastatic dormancy in breast cancer, see [15]; a quantitative assessment of the contribution of the above-defined states 1-3 of the metastatic cascade to the timing of metastatic relapse based on a mathematical model applied to real data can be found in [16]). Whether or not metastases will escape from dormancy in a particular patient depends not only on the effects of treatment, functioning of the immune system, concentrations of circulating angiogenesis promoters and inhibitors, and other internal factors; exacerbation of the disease may also be triggered by intercurrent sporadic external events such as surgery unrelated to breast cancer, infection, trauma, radiation, stress, etc. Another highly significant prognostic factor is the intrinsic aggressiveness of the disease; however, its reliable assessment at early stages of the disease has proven so far to be elusive. Thus, the most critical determinants of the trial outcome are largely unobservable and/or unpredictable.

In practice, the above unobservable prognostic factors are substituted with less informative observable surrogates such as (1) age at trial entry; (2) stage and histological grade of the disease at surgery; (3) localization and size of the primary tumor; (4) whether or not the tumor invaded surrounding tissues; (5) the extent of nodal involvement; (6) menopausal status; (7) estrogen and progesterone receptor status; (8) presence of specific mutations in BRCA1 or BRCA2 genes; (9) family history of breast cancer; and (10) individual history of other malignancies. Even this rough and incomplete set of surrogate clinical variables creates a large number of categories of women in both arms of the trial with potentially very different characteristics of survival and metastasis-free survival. Importantly, randomization won’t eliminate the observable and hidden heterogeneity; it will only reduce the difference in the extent of heterogeneity between the treatment and control arms.

The aforementioned inter-subject heterogeneity is quite typical of clinical trials (as opposed to in vitro experiments with cell lines or studies on animal models with tightly controlled inter-subject variation). Thus, individual responses of subjects in both arms of a trial cannot even approximately be viewed as homogeneous, let alone distributionally identical.

## Statistical inference from clinical trials: Are the assumptions met?

Like all mathematical sciences, theoretical statistics is based on theorems consisting of assumptions and conclusions. The validity of the arguments by which the conclusions are derived from the assumptions is there for anyone to verify. In applied statistics, including inference from clinical trials, statistical methods and tests resulting from these theorems are employed to generate new knowledge based on empirical data. But are the assumptions behind the methods and tests valid?

The most fundamental assumption that underlies virtually every application of statistics is that the set of observations, say x_1_, x_2_, …, x_n_, is a random sample from a certain probability distribution. Informally, this means that the observed values result from independent replications of the same random experiment, just like a sequence of “heads” and “tails” results from flips of a coin or numbers 1-6 result from repeated rolls of a die. The exact meaning of the “random sample” assumption is as follows: there exist a sample space S with a probability measure on it and jointly stochastically independent (i) and identically distributed (id) random variables X_1_, X_2_, …, X_n_ on S such that X_1_(s) = x_1_, X_2_(s) = x_2_, …, X_n_(s) = x_n_ for some point s in S. Critically, the iid assumption cannot be verified empirically, for each of these random variables is represented in the data set by a *single* value. The hypothesis that random variables X_1_, X_2_, …, X_n_ have a given distribution (say, standard Gaussian) can only be tested, with certain probability of error, under the iid assumption. Beyond this premise, most statistical methods, tests and tools fail; even measures as simple as the sample mean lose their inferential significance. Thus, the most basic assumption underlying statistical inference from data is necessarily and invariably taken on faith.

How strong is our faith in the iid hypothesis in the case of clinical trials? Turning to the independence property first, note that selection of trial participants is associated with clinical characteristics of their disease and other medical conditions and thereby constitutes a systematic source of dependence between individual response variables. The latter may also be induced by a pre-randomization run-in period during which all participants receive the same treatment [12]. Another factor is the significance of family history for the incidence of various health-related events, both sporadic and triggered by a medical intervention. For identical twins, the occurrence of such an event in one of them typically sharply increases the probability of the same event happening to the other twin, thus making these events highly dependent. The same effect, albeit possibly to a lesser extent, is often observed in siblings and other relatives. Furthermore, because in a relatively homogeneous human population it is very likely that two given members have a common ancestor, their disease states and responses to treatment should, at least in principle, be viewed as dependent random variables. Finally, stochastic dependence between individual responses may result from various post-randomization events such as exchange of information between subjects participating in a clinical trial, which may modify the placebo effect and lead to partial unblinding of the study.

One may argue, of course, that the aforementioned dependence is weak and therefore negligible. To analyze claims about the strength of dependence, the latter has to be quantified. A simple and almost universally used measure of dependence between two random variables is their correlation coefficient. This measure, however, is inadequate because (1) zero correlation does not imply independence; and (2) a collection of pairwise independent random variables may not be jointly independent. Thus, even designing a practicable quantitative measure of the deviation from joint independence represents a considerable challenge.

The central question in assessing the effects of stochastic dependence between individual response variables is how much the deviation from independence modifies the distribution of the test statistic under the null (no relative effect of intervention) and alternative (relative effect exceeding a given threshold) hypotheses. The distributions of the test statistics typically used in parametric analyses of clinical trial data (such as standard Gaussian, Student’s t and chi-squared) depend in very essential ways on the independence postulate. Absent this assumption, one cannot rely on these standard distributions anymore. An equally important question is how to assess the impact of misspecification of the distribution of the test statistic on the outcome of statistical analysis (such as the *p*-value of the test statistic under the null hypothesis, statistical power of the trial, sample size, estimates and confidence intervals for parameters of interest, trial stopping time, etc.).

As we have argued in the previous section, along with the possible lack of independence, statistical analysis of clinical trial data is bound to encounter an even more consequential violation of the equidistribution (id) assumption. As with stochastic dependence, to assess the extent of the deviation from the id assumption and its impact on the outcome of statistical analysis of trial data quantitatively, one has to use certain distance, d, between probability distributions. This can be done by employing one of the well-known probability metrics such as the total variation, Kantorovich, Kolmogorov-Smirnov, Cramér-von Mises, Lévy and other distances [17].

We emphasize once again that neither the absolute value, r, of the correlation coefficient for a pair of individual observations nor the distance, d, between their distributions is estimable from the observations alone. Suppose, however, for the purpose of our argument, that we know the values of r and d for all pairs of underlying random variables exactly and that they are small, say, less than some positive number ε. Let y be an output of statistical analysis. Computation of y is based on the known distribution, P_0_, of a test statistic under the null or alternative hypothesis provided the iid assumption is met. Let P be the “true” distribution of the same statistic under the hypothesis in question without the iid assumption. How much does P deviate from the “ideal” distribution P_0_? It can be envisioned that in some cases distribution P_0_ is robust, i.e. the distance d(P, P_0_) will be small for small ε, while in other cases d(P, P_0_) will be found to be large regardless of how small ε is. Similarly, the dependence of the output y on the “ideal” distribution P_0_ may be robust to the perturbations of the latter or not at all. Finally, even under the total robustness scenario, the utility of such a sensitivity analysis depends on availability of tight and relatively simple estimates for the deviation of the output y as a function of ε. Obtaining such estimates in most cases goes far beyond the reach of contemporary probability theory and statistics.

The patients’ hidden and observable clinical variables associated with the disease, responses to the compared interventions and susceptibility to the placebo effect partition the queried population into a large number of categories with distinct distributional characteristics of the response. Even if we assume that each category is distributionally homogeneous, both trial arms will contain a large yet unknown number of categories, each containing an unknown number of subjects. Moreover, the number of such categories in each arm and the numbers of their representatives are dependent random variables with unknown distributions. The unknown population weights of these categories are nuisance variables that will confound statistical analysis of trial outcomes. Furthermore, variation in the number of categories and subjects representing each category between different trials will make their results potentially irreproducible.

Theoretically, greater distributional homogeneity of responses in a clinical trial could be achieved through stratification with respect to observable clinical variables. However, two impediments will most likely undermine the feasibility of this approach. First, due to the large number of strata the requisite sample size in many individual strata will be unachievable, thus making the trial underpowered. Second, the presence of substantial hidden variation of the type discussed in the above breast cancer example will still leave individual strata heterogeneous.

In summary, even for large-scale randomized controlled clinical trials commonly viewed as the gold standard of biomedical research, the “heterogeneity curse” will likely make the results of statistical inference from the collected data dubious and potentially lead to false and irreproducible conclusions.

## Optimal sample size? Try *n* = 1!

One of the primary design parameters in a clinical trial is sample size. Large sample size is supposed to ensure statistical power of the study when the knowledge of causes and mechanisms of the underlying biomedical processes and effects of the compared interventions is insufficient for outcome prediction. As discussed above, such lack of knowledge likely means that, in spite of all the effort, the queried population is still heterogeneous and so is the sample of trial participants randomized between two or more arms of the study.

There is one case, however, where the heterogeneity and independence problems are non-existent, namely when a sample consists of just one subject. A great advantage of individual case studies is that learning everything that is there to know is quite feasible and that in this case inference from the generated data is not confounded by inter-subject variation. If one believes that biomedical processes are governed by natural laws and have causes, mechanisms and effects, then studying a single subject thoroughly should be very informative. It can be expected that doing so for many individual patients will eventually reveal all major types and characteristics of the disease and will enable evaluation, and even prediction, of effects, and side effects, of various interventions. However, in contrast to clinical trials, individual case studies is an open-ended process with uncertain inferential value, which makes them unfit for making expeditious public health decisions regarding medical interventions.

Since time immemorial medical doctors used the method of trial and error to find effective individual treatments while minimizing the harm to the patient’s health. In the cases where the attempted ineffective treatments did not change significantly the natural history of the disease, the patients served as best-matching self-controls. Multiple individual case studies, especially those involving controls, is the way medicine accumulated enormous empirical knowledge. Over the last two centuries, this process has been greatly accelerated by the advancement of basic biomedical sciences, and there is no reason to believe that it won’t bear fruit in the future. Focused on individual rather than population dimension of medicine, individual case studies represent a natural complement to clinical trials. Thus, before starting a clinical trial on 1000 patients, it is reasonable to ask if it would be more beneficial to science and health care (as well as more cost-effective) to conduct a more sophisticated, state-of-the-art individual case-controlled study on 100 subjects randomly selected from a larger pool of qualifying and consenting patients.

## Statistical analysis of clinical trials as a ritual

Distributional heterogeneity of responses within the queried population and potential lack of independence are by no means the only factors that may call into question the results of statistical inference from trial data. Statistical analysis of clinical trials involves a whole host of hidden and untestable assumptions, various approximations and arbitrarily selected thresholds discussed below. They all require careful justification and thorough theoretical, or at least numerical, sensitivity analysis. Without this, statistical inference from clinical trials would essentially be a ritual that lacks rigorous scientific underpinnings and may have disastrous effects on public health.

### The mantra of large n and the invocation of normality

The distributions of the test statistics most widely used in the analysis of clinical trials are the standard Gaussian (normal) and closely related distributions (such as χ^2^ and Student’s t). Strictly speaking, their legitimacy is contingent on the assumption that individual response variables are iid with normal distribution. Recall that the normality ﻿assumption is empirically untestable without the iid hypothesis that, as we have argued above, can by no means be taken for granted. The iid assumption is also required if one employs asymptotic results such as the Central Limit Theorem that allows one to conclude that for large sample size the distribution of the sample mean of the response variables over a trial arm is approximately normal. An important question is how large the sample size should be for the true finite sample distribution be sufficiently close to the asymptotic distribution. Estimates of various distances between these distributions as function of sample size are extremely hard to obtain. Even for the Central Limit Theorem, only a basic estimate of the Kolmogorov-Smirnov distance given by the Berry-Esseen theorem [18] is available. Yet another difficult question concerns the effects of such estimates on the accuracy of the outcomes of statistical analysis.

Additional challenge to the normality assumption comes from the obvious fact that individual response variables, and hence their sample means, are bounded above and below, so that their true distributions are always confined to a finite interval; in particular, they can never be exactly normal, a point eloquently made in [12]. The correction to the assumed asymptotic distribution with an infinite tail that arises from such a truncation, as measured by some probability metric, may be small; however, the downstream effects of this error on the distribution of the test statistic and outcomes of statistical analysis may be significant.

### Sample size: Fixed or random?

Although statistical analysis of trial data assumes fixed sample size, it is often applied to sample size that is in reality random. Variation of the sample size has several sources. One is randomization of patients between the trial arms; here, sample size variation in each arm may be substantial unless block randomization or more advanced schemes [19, 20] are employed. Random sample size also arises in trials that require a fixed number of *events* of interest. Finally, many patients drop out of a trial due to the lack of benefit, severe side effects or other reasons, which leads to a difficult dilemma: either to perform “intent-to-treat” analysis with fixed sample size under intractable informative censoring or to deal with a random number of patients followed through the entire study duration. Statistical methods intended for fixed sample size lead to erroneous results if applied to samples of random size. For example, even the Central Limit Theorem that underpins many statistical methods fails beyond a few special cases, e.g. when the sample size has Poisson distribution ([21], p. 4699).

### The idol of statistical significance

Statistical significance emerged as a means to account for random variation in the context of hypothesis testing. Let, for example, δ be the observed value of the difference Δ = A_1_ – A_0_ between the average measures of effect for experimental and control arms of a clinical trial. Is the observed relative effect due to the true difference between the compared interventions or to chance, or perhaps both? A reasonable question to ask, then, in order to distinguish between these two possibilities, is as follows: What is the probability to observe the value δ under the (strong) null hypothesis that, for each trial participant, the effects of the compared treatments are identical? In other words, what is the probability that Δ = δ due to chance alone? If the distribution, P, of the statistic Δ under the null hypothesis were discrete, then the required probability would be P(δ). The problem with this answer, still somewhat popular among natural scientists, is that it does not provide a clear way to separate two interrelated factors: (1) the magnitude of P(δ) relative to the probabilities P(x) of other admissible observations x; and (2) the sample size n. (Note that as n increases all the probabilities P(x) tend to become small). In the opposite case of a continuous distribution P, the proposed answer is utterly uninformative, for in this case P(x) = 0 for any observation x.

A way to resolve this conundrum was proposed by Sir Ronald Fisher in his famous book [22]. To quantify significance of an observation δ, he suggested to use the probability, under the null hypothesis, that Δ ≥ δ if δ > 0 and Δ ≤ δ if δ < 0 (or the corresponding two-tail probability if the sign of Δ is of no particular importance). This probability, termed the *p*-value, represents the asymptotic fraction of hypothetical independent identical trials, if one conducts them indefinitely, in which the size of the observed effect will be at least as extreme as that in the given trial. Thus, sufficiently small *p*-values can be used for rejecting the null hypothesis.

Fisher’s approach to significance is not without a blemish. First, it employs, contrary to the empirical nature of biomedical sciences, the values of statistic Δ that were not observed in a given study and perhaps will never be, even if the study were to be replicated indefinitely; the sole basis for these counterfactual values of Δ is its imputed distribution under the null hypothesis. Second, Fisher’s idea is based on a tacit assumption that the probability density function of statistic Δ under the null hypothesis has a one- or two-sided bell-shaped tail. For other shapes, it may lose its appeal (think, for example, of Δ uniformly distributed on a symmetric interval whose endpoints represent realistic bounds for Δ). Furthermore, if the null distribution of Δ is multimodal then the blanket definition of *p*-value as tail probability is unequivocally wrong.

Normal distribution of Δ, almost universally assumed in the parametric analyses of clinical trials, is merely an approximation, based on the Central Limit Theorem, to its true distribution (or perhaps not even an approximation if individual response variables are not iid). Because *p*-value is a tail probability, the resulting error in its determination may be as large as the Kolmogorov-Smirnov distance between the two distributions. The latter can be estimated through the Kolmogorov-Smirnov distances between the distributions of the averages A_1_, A_0_ over trial arms and their normal approximations. Under the iid assumption, each of these distances, according to the Berry-Esseen theorem [18], does not exceed 0.5Cn^-1/2^, where n is the sample size and C ≥ 1 is the ratio of the third absolute central moment of the distribution of individual response variables to the cube of its standard deviation. Importantly, the dependence of the Berry-Esseen bound on n cannot be improved; specifically, with the upper bound of 0.4Cn^-1/2^, it is in general not true anymore [23]. Therefore, for sample sizes typically encountered in clinical trials (from a few hundred to a few thousand subjects), the maximum error in *p*-value determination may be comparable to, or even exceed, the small *p*-values used for rejecting the null hypothesis. Such sample sizes can only *guarantee* the correctness of the *first* decimal digit of the *p*-value! Thus, pursuit of small *p*-values in parametric analysis of clinical trials is indefensible. A way to somewhat mitigate this problem is discussed next.

### Randomization ignored

The above-described parametric *p*-values ignore randomization, an essential aspect of the design of randomized controlled clinical trials [12]. Suppose that *p*-values are computed under the (strong) null hypothesis. Then, for a properly blinded trial, it is reasonable to expect the responses of all trial participants to be *exactly the same* regardless of their allocation to treatment arms (this claim is unequivocally true if individual responses are deterministic). This enables computation of the statistic Δ for *every possible* allocation of subjects to treatments that the employed randomization algorithm may produce, not only the “real” one. This leads to the permutation-based *p*-value of the observation δ [24, 25]. If individual responses are deterministic (in which case stochastic dependence and heterogeneity are non-issues), this is *the only way* to compute the effective significance of the trial outcome. However, in general these *p*-values are only partial in that they do not account for the variation of individual response variables. This is where parametric analysis, that capitalizes on the asymptotic normality of Δ, could be invoked. (Observe that because the generalized Berry-Essen inequality [26] requires only independence of individual response variables, the error in *p*-value determination can in principle be controlled even if the assumption of distributional homogeneity is lifted). The average of parametric *p*-values computed over all admissible outcomes of the randomization process represents a permutation-based parametric *p*-value that will most likely reduce the error in the conventional parametric *p*-value computation.

### The magic 5% and other arbitrary thresholds

In the aforementioned book [22], Ronald Fisher also proposed to use 0.05 as a *p*-value threshold for rejecting the null hypothesis. Since then this low key suggestion has become almost a religious commandment for adoption of statistical significance levels and computation of confidence intervals. For example, a recent massive meta-analysis study [27] found that among almost 2 million biomedical papers published over the last 25 years, 96% appealed to *p*-value ≤ 0.05 to claim significance of their results. As discussed above, numerous factors may lead to misspecification of the test statistic under the null hypothesis, which may have a considerable impact on the effective significance level of a clinical trial. As a result, a trial may produce false results even if the *p*-value happens to be < 0.05 and, conversely, true and valuable results may be discarded or self-censored just because their statistical significance falls short of the magic 5%. The same is true for the deviation of the effective statistical power of clinical trials under the alternative hypothesis from the nominal value, typically assumed at the planning stage to be 80 or 90%.

## Discussion, conclusions and recommendations

That logic, including careful formulation of premises, is critical for the correctness of all sorts of arguments, has been recognized since Aristotle. However, it was Henri Poincaré, a genius French mathematician, theoretical physicist and philosopher of the 19th and early twentieth century, who was the first to keenly understand the fundamental importance of hypotheses and assumptions for the validity of scientific research [28]. Apparently, his ideas appeared so much ahead of their time that even more than a century later they have not been fully recognized and taken to heart by the scientific community.

In this work, we focused on the following basic assumptions that underpin statistical methodology used in clinical trials and are critical to the validity of statistical inference from trial data: (1) substantial role of intra-subject variation in individual responses to the compared interventions; (2) stochastic independence of individual response variables; and (3) their distributional homogeneity within each trial arm. We found that for many health conditions and treatments assumption 1 is unlikely to be true; assumption 2 is possibly approximately true with some exceptions and in general requires careful analysis in each particular case; and assumption 3 is likely to be false. The last point has two important implications.

- The average response over a trial arm may prove to be a poor preference function for selection of the best intervention and likewise the normalized difference between arm-specific averages may appear to be a suboptimal test statistic under the null and alternative hypotheses. As a historical note, the idea that indiscriminant use of averages in biology and medicine may lead to obfuscation of scientific truth was passionately argued 150 years ago by Claude Bernard, one of the greatest experimental physiologists of all times. He also insisted that the duty of a scientist is to find the unique immediate cause behind every health-related event in an individual patient, thus siding unequivocally with the deterministic paradigm ([29], p. 137).
- Under the assumption of stochastic individual responses, a key condition that makes or breaks statistical analysis of clinical trial data is the distributional homogeneity of individual response variables. On the one end of the homogeneity spectrum, one encounters the situation where individual response variables in each arm of the trial are iid. Here comparison of treatments by the value of arm-specific averages and inference from their difference, when made correctly and rigorously, is well justified. Achieving a larger degree of homogeneity requires tightening of the subject recruitment criteria based on observable clinical variables including genomic, molecular, cellular, histologic and other markers of the disease and responses to compared treatments. Nonetheless, it is quite possible that in spite of all the effort, responses of trial participants are still extremely heterogeneous. There we meet the other end of the homogeneity spectrum where all individual responses have very dissimilar distributions. Here each individual subject represents a separate clinical case while population approach has only descriptive utility. Each combination of a disease, medical intervention and targeted population falls somewhere in between these extremes but its position within the homogeneity spectrum is difficult to determine. A practical way to address this uncertainty would be to conduct clinical trials and controlled individual case studies competitively and compare their outcomes.

From its early beginnings medicine has been “personalized” in that physicians were primarily focused on treating individual patients, one at a time, and their interventions were tailored to the unique patient’s condition and specific course of the disease. The advent of clinical trials with its focus on average population effects signified a dramatic departure from this paradigm. One conclusion of this work is that perhaps the time has come to expand the approach of personalized medicine from treatment to drug development and other medical innovations. Due to scientific and technological breakthroughs in molecular biology and genomics combined with increased computational power and modern information technologies, such expansion may bring about greater effectiveness and prognostic accuracy of medical treatments than in the past, see e.g. [5].

In this work, we have also taken a close look at statistical significance invoked for supporting the claim of superiority of a tested intervention over a comparator. Many factors, such as (1) violation of the iid assumption for individual responses; (2) deviation from the normality of the individual responses or their arm-specific averages; (3) randomness of the sample size; and (4) failure to take into account randomization of trial participants between treatments, may cause the *p*-value computed for the postulated distribution of the test statistic to diverge considerably from the true significance. For example, as we have argued in the previous section, to *guarantee* the nominal significance α = 0.05 (after rounding) in parametric analysis, a trial has to be run with tens of thousands subjects! This casts a serious doubt on the utility of parametric *p*-values for planning and analyzing clinical trials, suggests that small parametric *p*-values are likely meaningless, and implies that reliance on fixed thresholds (typically 0.05) for rejection of the null hypothesis is scientifically unfounded. The last point is also true for statistical power. Finally, the conventional sample size computation at the planning stage of a clinical trial may also lead to erroneous results.

To make things even worse, in the vast majority of biomedical studies (including reports on clinical trials), *p*-values are deployed without even defining the measure of effect, stating the null hypothesis or specifying the test statistic, let alone verifying the assumptions under which the test statistic has a postulated distribution. As a result, systematic misuse, overuse and misinterpretation of *p*-values has become a major source of false and irreproducible results. Although these abuses have been extensively criticized [30–33], *p*-values remain the single most important numerical measure invoked to analyze the results of clinical trials, confirm the validity of biomedical research, and make critical health care decisions. Yet all too often they provide a convenient cover for poor data quality, all sorts of biases and conflicts of interest pertaining to the collection, analysis and reporting of clinical trial data, and for outright fraud.

Throughout history the practice of medicine was rooted in tradition, authoritative opinion, personal experience, and clinical intuition. Clinical trials emerged as an attempt at a more “objective” and “evidence-based” approach. If a treatment has invariably large effect then a small controlled trial would give a definitive answer even without formal statistical analysis. A textbook example is the finding that eating citrus fruits or drinking their juice cures scurvy, a discovery made in 1747, long before it was found that scurvy is caused by vitamin C deficiency, by Royal Navy surgeon James Lind through the first controlled trial in history. As Sir Austin Bradford Hill has forcefully stated in his classic work [34], in cases where the observed effect is uniformly very large, very small or practically inconsequential, formal statistical analysis is unnecessary. It is the case of heterogeneous effects of small or variable size that calls for carefully designed large-scale controlled randomized clinical trials and rigorous statistical analysis of their outcomes. Paradoxically, as we have argued in this work, this is precisely the situation where distributional heterogeneity of individual responses and numerous other factors may invalidate the basic assumptions upon which statistical analysis of clinical trial data rests and result in false and irreproducible conclusions.

What should be done to restore the value and integrity of statistical methods in clinical trials and beyond? I believe the answer lies in (1) resisting statistical orthodoxy and creatively using a multitude of statistical methods (while occasionally openly admitting that these methods have failed); (2) rigorously validating all the assumptions underlying statistical analysis; and (3) closely coordinating statistical analyses with biomedical research, which provides statistical methods with both context and means of external validation. As an example of how much enrichment, innovation and modification biomedical research may bring to statistical methodology, the reader is referred to the article [35], which represents a twenty-first century version of the Bradford Hill’s 9 principles of inference from association to causation in epidemiological studies that he has formulated in 1965 [34]. Each clinical trial should be treated as a unique scientific project that brings the full arsenal of knowledge of mechanisms associated with the disease and its treatments to bear on selection, or invention, of statistical methods. Meanwhile, advanced mathematical and computational methods could be used to validate the assumptions behind statistical methods and estimate errors resulting from various approximations.

We conclude with a few specific recommendations informed by the analysis undertaken in this work.

1. Clinical trials should be publicly funded and conducted by biomedical researchers, medical doctors and statisticians with no relation to industry and no conflicts of interest.
2. Health care decisions based on outcomes of clinical trials should rely on a combination of statistical and biomedical evidence.
3. Scientific and health care benefits resulting from clinical trials should be compared to those of state-of-the-art controlled individual case studies incurring comparable costs.
4. Trials should be populated in such a way that the anticipated individual responses in all arms of the trial are as homogeneous as possible given all the available prior information.
5. Results of statistical analyses of randomized clinical trial data should be compared with those based on deterministic individual responses and permutation-based *p*-values, unless there is strong scientific evidence that individual responses are stochastic.
6. The use of fixed levels of significance and statistical power as well as pursuit of small *p*-values in parametric analyses of trial data should be discouraged.
7. Computation of parametric *p*-values for randomized clinical trial data should involve averaging over the set of permutations produced by the randomization algorithm.
